# Variants in Nucleotide Sequences; Gene Expression; and Hematological, Immune, and Antioxidant Biomarkers Linked to Pneumonia Risk in Holstein Calves

**DOI:** 10.3390/vetsci12070620

**Published:** 2025-06-26

**Authors:** Ahmed El-Sayed, Attia Eissa, Doaa Ebrahim, Ahmed Ateya, Hossam Gadalla, Hanan M. Alharbi, Khairiah M. Alwutayd, Manal A. Babaker, Aya Aly Elzeer

**Affiliations:** 1 Department of Animal Health and Poultry, Animal and Poultry Production Division, Desert Research Center (DRC), Cairo 11753, Egypt; 2 Department of Animal Medicine (Internal Medicine), Faculty of Veterinary Medicine, Arish University, Arish 45511, Egypt; attia.ahmed@vet.aru.edu.eg; 3 Department of Respiratory Center, College of Applied Medical Sciences-Jubal 4030 (CAMSJ), Imam Abdulrahman Bin Faisal University, Al Jubail 38516, Saudi Arabia; arabia.daradwan@iau.edu.eg; 4 Department of Development of Animal Wealth, Faculty of Veterinary Medicine, Mansoura University, Mansoura 35516, Egypt; yoyo_elzeer@mans.edu.eg; 5 Department of Clinical Pathology, Faculty of Veterinary Medicine, Mansoura University, Mansoura 35516, Egypt; gadallaha@mans.edu.eg; 6 Department of Biology, College of Science, Princess Nourah bint Abdulrahman University, P.O. Box 84428, Riyadh 11671, Saudi Arabia; hmalharbi@pnu.edu.sa (H.M.A.); kmalwateed@pnu.edu.sa (K.M.A.); 7 Department of Chemistry, Faculty of science, Majmaah University, Al Majmaah 11952, Saudi Arabia; m.babaker@mu.edu.sa

**Keywords:** antioxidants, calves, hematology, interleukin, genetic polymorphisms, pneumonia, gene profile

## Abstract

Pneumonia is a major issue that affects calves’ health and performance and causes numerous losses despite treatment. Investigating genetic and molecular differences, as well as immunological and antioxidant responses, in calves at risk for pneumonia was the aim of this study. A total of 225 calves were studied, including 180 Holstein calves with respiratory signs and 45 calves that were apparently healthy. Blood samples were collected for CBC, RNA extractions, and immunological and antioxidant analysis. In contrast to the control group, the pneumonic one showed a considerable (*p* < 0.05) increase in the expression levels of cytokines and antioxidant genes IL1α, IL-1β, IL-6, IFN-γ, TNF-α, and NOX4. In contrast, the values of IL10, PRDX6, ATG7, and NDUFS6 were in the opposite range. The pneumonic and healthy calves were found to differ in the nucleotide sequences of the genes under analysis. In pneumonic calves, a substantial (*p* ˂0.05) reduction was detected in RBCs, Hb count, PCV%, and lymphocytes count, and a notable (*p* ˂ 0.05) increase in WBCs and neutrophil count was correlated with healthy control calves. The findings of the serum profile showed that there was a meaningful (*p* ˂ 0.05) rise in the serum values of IL-1α, IL-1β, IL-6, TNF-α, IFN-γ, and MDA, with significant reductions in the SOD, GSH, TAC, and IL-10 in the pneumonic compared to the healthy calves. Our results provide valuable information about the nucleotide sequence, gene expression, and serum profile differences of putative indicators for pneumonia in calves. This could be applied in monitoring calves’ pneumonia through the discriminate breeding of naturally resistant animals.

## Simple Summary

Pneumonia is a serious problem that influences the health and productivity of calves and results in various losses. This work aimed to examine the immunological and antioxidant responses, as well as genetic and molecular differences, in calves that are prone to pneumonia. Out of the 225 calves, 180 Holstein calves had respiratory symptoms, while 45 cases seemed to be healthy. Blood was collected from the diseased and apparently healthy calves for CBC and RNA extraction. The pneumonic calves had significantly different hematological, immunological, and antioxidant blood levels; expression patterns; and SNPs of genes related to immunity and antioxidants when compared to healthy calves. These findings offer a practical strategy for reducing the incidence of pneumonia in calves by selective breeding based on genetic markers.

## 1. Introduction

Respiratory disease syndrome, which is a prevalent ailment among calves in all of the major nations that produce calves, is brought on by the lungs’ bronchioles and alveoli responding inflammatorily to infectious agents, causing pulmonary consolidation [1,2]. It is thought of as a complex disease that involves interactions between the host (physiological and immunological), several agents (bacterial, viral, and parasitic), nutrition, stress, and environmental factors [3,4]. It results in significant financial losses, including decreased growth performance, medical costs, malfunctioning reproductive organs, early culling, and death of sick calves [5]. Conferring to [6], lung resistance mechanisms are debilitated and illness can progress when a minimum level of infectious agents, host susceptibility, and non-specific resistance mechanisms is extended. As the size of the herd grows, the disease also grows. It is more common during the growing season, particularly in animals that are one to three months old [7]. Calves that are raised in cramped quarters and have environmental respiratory issues are also more likely to experience this condition. Crowding in barns with inadequate ventilation and the buildup of urine and manure cause the air in the barn to become heavily contaminated with ammonia, which severely irritates the mucous membrane of the upper respiratory tract and increases the risk of inflammation [8]. The erratic weather and drastic temperature swings between day and night make calves more susceptible to respiratory illnesses in the early spring and autumn [9]. According to [10,11], respiratory diseases cause a morbidity rate of roughly 30–65% per year and a mortality rate of 10–31% in calves. The main symptoms in infected calves include fever, coughing, abnormal breath and tear production, and abnormal lung auscultation sounds [12]. The complicated mechanisms of pneumonia development make prevention difficult even with advances in vaccine technology [13,14]. Numerous metabolite markers found by [15] were in line with earlier research, indicating that metabolomics could be helpful as a means of confirming bovine respiratory disorders (BRDs) in feedlot cattle after the first visual diagnosis.

An assessment of the seriousness of the injury to the body’s tissues could be made by detecting a deviation of some blood standards from their regular ranges [16]. An imbalance among antioxidants and oxidants, like free radicals or reactive oxygen species, frequently results in oxidative stress. However, through defense mechanisms, animal bodies can control excess free radicals [17,18].

Tumor necrosis factor (TNF), IL1, IL-6, IL-8, and other pro- and anti-inflammatory cytokines are released by the body during a natural infection or following vaccination [19,20]. In addition to attracting neutrophils and monocytes to the infection place, these cytokines are essential for promoting the production of acute-phase proteins, which are critical during acute inflammation [19]. Nonetheless, proinflammatory cytokines, including IL-1β, IL-8, and TNF-α, demonstrated 90% (68.3–98.8%), 100% (83.2–100%), and 85% (62.1–96.8%) diagnostic precision, respectively, in feedlot cattle, signifying that their application to BRD diagnosis may be possible [21]. A prior study likewise indicated that a combination of cytokines and APPs at arrival could aid in the development of a guide that can be used to more precisely identify animals that are more prone to having BRD [22].

Modern molecular genetic approaches are employed as a supplement to treat the disease and improve animal health [23]. Researchers have successfully reported genetic markers and primarily single-nucleotide polymorphisms (SNPs) for cattle disease susceptibility or resistance [24,25,26]. It implies that variations exist in the adaptability or resilience of host genomes to the disease [27].

Studies on the serum levels of proinflammatory cytokines and antioxidants and their possible application in the early diagnosis of pneumonia infection in calves are relatively scarce. In addition, no comprehensive investigation of the relationship between SNPs and altered gene expression, increased production of proinflammatory cytokines, and antioxidant response in pneumonic calves has been conducted. We hypothesize that the proinflammatory cytokines and antioxidant biomarkers can serve as diagnostic and monitoring tools for pneumonia in young calves [28]. Consequently, the objective of this work was to investigate the association between calf pneumonia and SNPs, gene expression, the serum profile of immunological and antioxidant indicators variations, and further hazard issues.

## 2. Material and Methods

### 2.1. Animals and Study Design

A total of 225 male Holstein calves with an average age of 3.1 ± 0.9 months (1.6–4.5 months) and average body weight of 74.7 ± 37 kg (30–140 kg), from eight different farms in Siwa Oasis, Egypt (lat., 29°06″ 29°24″ N; long., 25°16″ 26°12″ E), situated 330 km southwest of the Mediterranean coastline and 65 km east of the Libyan boundaries, were involved in the present study, from December 2023 to May 2024.

A systematic clinical examination was made [10]. All calves were evaluated for animal posture (normal vs. low head), mental status (alertness vs. unawareness), appetite (normal vs. appetite loss), rectal temperature (38.8 vs. 41.3 °C), pulse (93.8 vs. 145.4 beat/min), respiration rates (29.4 vs. 51.9 beat/min), nostril and eye secretion (presence vs. absence), and pulmonary auscultation (normal vs. abnormal lung sounds as crackles and wheezes). According to the clinical examination, calves were allocated into two groups. The control group (CON; n = 45) consisted of clinically healthy calves without presenting signs of pneumonia, and the pneumonic group (PG; n = 180) consisted of calves that were naturally infected and exhibited a range of clinical signs. The vaccination status of the calves and dam was undetermined. The calves lived in semi-open sheltered pens and were fed on a basal diet prepared for beef cattle [29]. The basal diet comprised 40% wheat straw and 60% concentrate. Free water was offered, along with a diet twofold a day in the morning and evening.

### 2.2. Blood Sampling

Jugular venipuncture was performed to collect 10 mL of blood from every calf immediately after clinical diagnosis. The blood was alienated into two types of collection tubes: EDTA-containing tubes for whole blood, and plain tubes without anticoagulants for serum separation. Immediately after collection, all samples were placed on crushed ice and promptly carried to the laboratory for supplementary processing. EDTA-treated whole blood was used for complete blood count (CBC) analysis and RNA extraction. Plain blood tubes were left overnight at room temperature, and then they were centrifuged at 3000 rpm for 15 min. The separated sera were fractionated, frozen at –20 °C, and stored for following immunological analysis.

### 2.3. Hematological Parameters

Hematological parameters, including red blood cell count (RBCs), hemoglobin concentration (Hb), packed cell volume (PCV), mean corpuscular volume (MCV), mean corpuscular hemoglobin (MCH), mean corpuscular hemoglobin concentration (MCHC), total white blood cell count (WBC), neutrophil count, and lymphocyte count, were measured using an automated hematology analyzer (Exigo EOS Veterinary Hematology System, Boule Medical AB, Domnarvsgatan 4a 163 53, Spånga, Sweden).

### 2.4. Total RNA Extraction, Reverse Transcription, and Quantitative Real-Time PCR

In accordance with the manufacturer’s references, overall RNA was isolated from calf blood with TRIzol reagent (RNeasy Mini Ki, Catalogue no. 74104, Qiagen Inc., Germantown, MD, USA). The NanoDrop^®^ ND-1000 Spectrophotometer was used to quantify and qualify the amount of isolated RNA. Each sample’s cDNA was created in accordance with the manufacturing process (Thermo Fisher, Waltham, WA, USA, Catalog no, EP0441). Using quantitative RT-PCR with SYBR Green PCR Master Mix (2× SensiFastTM SYBR, Bioline, CAT No: Bio-98002), the gene expression patterns for coding segments of genes encoding immunity (IL-1α, IL1B, IL6, TNF-α, IL10, and IFN-γ) and antioxidant (PRDX6, ATG7, NDUFS6, and NOX4) were evaluated. Via real-time PCR with the SYBR Green PCR Master Mix (Quantitect SYBR green PCR kit, Catalogue no. 204141, Qiagen, Hilden, Germany), the relative mRNA level was measured.

Primers were created based on the *Bos taurus* nucleotides that was issued in PubMed, as indicated in Table 1. As a constitutive control for normalization, the housekeeping gene GAPDH was employed. The reaction mixture, which had a total volume of 25 µL, contained 3 µL of total RNA, 4 µL of 5× Trans Amp buffer, 0.25 µL of reverse transcriptase, 0.5 µL of each primer, 12.5 µL of 2× Quantitect SYBR green PCR master mix, and 8.25 µL of RNase-free water. After putting the finished reaction mixture in a thermal cycler, the following procedure was run: reverse transcription for 30 min at 50 °C; primary denaturation for 10 min at 94 °C; 40 cycles of 94 °C for 15 s; annealing temperatures for 1 min, as indicated in Table 1; and 72 °C for 30 s. A melting curve analysis was conducted at the conclusion of the amplification step to verify the PCR product’s specificity. Using the 2−ΔΔCt technique, the relative expression of each gene per sample was compared to that of the GAPDH gene [30].

### 2.5. DNA Sequencing and Polymorphism Detection

Removal of primer dimmers, nonspecific bands, and other contaminants was performed prior to DNA sequencing. As described by [31], a PCR purification kit was used in accordance with the manufacturer’s instructions to purify real-time PCR yields with the desired size (target bands) (Jena Bioscience # pp-201×s/Jene, Germany). The Nanodrop (UV-Vis spectrophotometer Q5000/USA, NanoDrop Technologies, Wilmington, DE, USA) was used to quantify the PCR product for producing superior results and guarantee sufficient concentrations and transparency of the PCR yields [32]. PCR results containing the target band were sent for forward and reverse DNA sequencing using an ABI 3730XL DNA sequencer (Applied Biosystem, Foster City, CA, USA) in order to identify SNPs in genes examined in control and pneumonia-affected calves. This was performed using an enzyme-chain terminator technique created by [33]. Chromas 1.45 and blast 2.0 tools were used to analyze the DNA-sequencing data [34]. PCR results of the genes under investigation and reference sequences found in GenBank were compared, and differences were categorized as single-nucleotide polymorphisms (SNPs). Based on DNA sequencing-data alignment, the MEGA6 software tool was used to compare the amino acid sequences of the genes under investigation among enrolled calves [35].

### 2.6. Inflammatory and Antioxidant Biomarkers

Serum immunological investigations were performed by profitable test kits, following the manufacturers’ typical protocols. The subsequent kits were used to enumerate the serum level: IL 1 α, IL 1 β and IFNᵧ ELISA Kit (Ray Biotech, Inc., CAT No: ELB-IL1a-1 and ELH-IL1b-1 and ELB-IFNg-1, respectively); IL 6, IL10 (BOSTER BIOLOGICAL TECHNOLOGY, CAT No: EK0412 and EK0418, respectively), and TNF-α ELISA Kit (AVIVA SYSTEM BIOLOGY); and malondialdehyde (MDA), antioxidants (super oxide dismutase (SOD), glutathione reduced (GSH), and total antioxidant capacity (TAC) (spectrophotometrically, using commercial kits from Biodiagnostic Company^®^, Giza, Egypt).

### 2.7. Statistical Analysis

The SPSS software version 23 was utilized to compare the measured variable means of the PG and CON groups utilizing independent-samples *t*-test. The PG and CG variables were all displayed as mean ± SD. The correlations between the genetic and immunological factors were determined using Pearson’s simple correlation test. Difference in the frequencies of each gene SNP between pneumonic and healthy calves was statistically assessed using Chi-square test to match the distribution of the recognized SNPs among the two groups, using SPSS version 23, USA. A Linear Discriminant Analysis (LDA) was conducted to determine whether gene-level SNP averages could be used to differentiate between pneumonic and healthy calves. The 21 genes’ average scores served as predictor variables, and the health status (pneumonic vs. healthy) was the grouping variable. A level of *p* = 0.05 was considered significant for all tests.

## 3. Results

### 3.1. Clinical Examination and Hematological Profile

In comparison to clinically healthy calves, pneumonic calves displayed a wide range of the greatest common detectable clinical symptoms of respiratory disease syndrome and a marked increase in body temperature (41.3 ± 0.3 °C), respiratory rate (145.4 ± 4.5 beat/min, and pulse rate (51.9 ± 2.3 breath/min) (Table 2).

Lower (*p* = 0.001) RBCs, Hb count, PCV, and lymphocytes (*p* = 0.03) count; and higher WBCs (*p* = 0.001) and neutrophil (*p* = 0.004) counts were observed in the PG than in the CON group (Table 3).

### 3.2. Patterns for Transcript Levels of Immune Indicators

**Figure 1:**
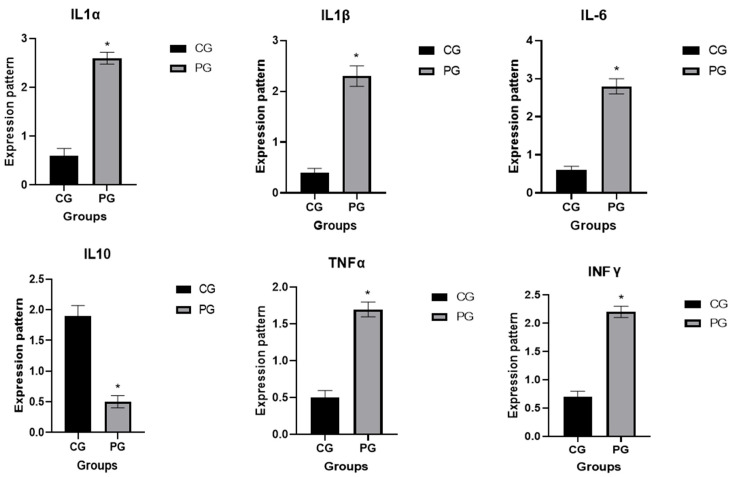
Normal (CG) and pneumonia-affected caves (PG) have different immune gene transcript levels. When *p* is less than 0.05, significance is showed by the sign *: *p* < 0.05.

**Figure 2:**
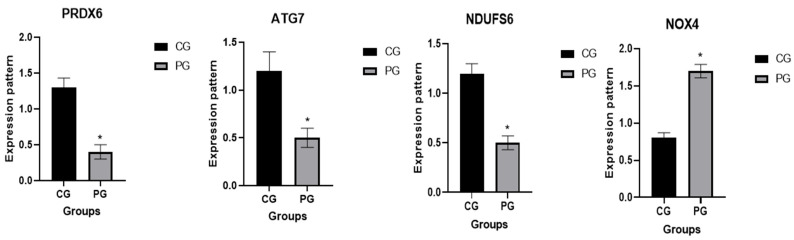
Normal (CG) and pneumonia-affected caves (PG) have different antioxidant gene transcript levels. When *p* is less than 0.05, significance is directed by the mark *: *p* < 0.05.

The transcription patterns for the valued immunological and antioxidant markers are shown in Figure 1 and Figure 2, respectively. The genes IL-1α, IL1B, IL6, TNF-α, IL10, IFN-γ, and NOX4 had significantly greater gene expression levels in pneumonia-affected calves compared to the CON group. Meanwhile, the levels of ATG7, NDUFS6, PRDX6, and IL10 dropped.

Pneumonic and healthy calves were compared for the highest and lowest levels of expression of immunological and antioxidant genes. The utmost potential level of mRNA for pneumonic calves was 2.82 ± 0.21 for IL-6, whereas the lowest extent of each marker was 0.45 ± 0.12 for PRDX6. Among all the genes tested in the CON group, IL-10 had the highest possible value of mRNA (1.96 ± 0.17), whereas IL1B had the least quantity (0.49 ± 0.08).

### 3.3. Genetic Polymorphisms of Immune and Antioxidant Genes

Using PCR-DNA sequence verdicts, the IL-1α (356-bp), IL-1β (423-bp), IL-6 (384-bp), TNFα (490-bp), IL-10 (306-bp), IFN-γ (321-bp), PRDX6 (434-bp), ATG7 (416-bp), NDUFS6 (405-bp), and NOX4 (396-bp) genes were found to have different SNPs in the amplified DNA bases linked to pneumonia. All of the detected SNPs were authorized based on DNA sequence variances between immunological and antioxidant markers tested in the calves under research, as well as reference gene sequences obtained from GenBank (Figures S1–S10).

The coding DNA sequences of the affected calves differed from those of the healthy calves due to the exonic-region abnormalities that are seen in Table 4 in all of the immunological and antioxidant markers tested. Using DNA sequencing of immune and antioxidant genes, 22 SNPs were found. Eight of them were non-synonymous, and fourteen of them were synonymous. Six of the eight non-synonymous SNPs were associated with healthy calves, while two were associated with pneumonic calves. The incidence of pneumonia, on the other hand, was linked to seven of the fourteen synonymous SNPs revealed.

Two of the recurrent SNPs found by DNA sequencing for the immunological markers of the IL-1α gene (356-bp) were G56C and A182G, which resulted in non-synonymous alterations S19R and K61R, respectively. The 100N amino acid, on the other hand, was identified as the consequence of the synonymous mutation T300C. The IL-1β gene’s 423 bp DNA sequence was examined, and one recurrent SNP—C153T—was found to carry the synonymous mutation 51I. The IL-6 (384 bp) gene was sequenced, and two repeated SNPs were discovered. S74N was a non-synonymous mutation in G221A, while R108K was involved in G323A. DNA sequencing revealed that the IL-10 gene contains four regular SNPs (306 bp); three of them, A105T, G129C, and C168T, corresponded to synonymous alterations of 35S, 43L, and 56C, respectively. The G203C SNP, meanwhile, resulted in the non-synonymous mutation R68K. Located in the 490 bp DNA structure of the TNFα marker, three frequently occurring synonymous SNPs—A120G, C213T, and T468C—were associated with amino acids 40Q, 71D, and 156Y, respectively. The IFN-γ gene’s 321 bp sequencing revealed one frequent SNP, where the G35T variation initiated the non-synonymous mutation G12V.

According to antioxidant markers, DNA sequencing revealed that the PRDX6 gene (434 bp) includes two recurrent SNPs that resulted in synonymous mutations, 39N and 75D, respectively. Three SNPs were trapped within the 416 bp AT7G gene. Two SNPs, T48C and T333C, were synonymous and connected to the amino acids 16S and 111A, respectively. Meanwhile, the amino acid V72I was generated by the non-synonymous SNP G214A. A synonymous SNP was found in the NDUFS6 gene’s nucleotide sequence (405 bp); the G354A SNP produced the 118S result. Sequencing the NOX4 (405 bp) gene revealed two recurrent SNPs. Non-synonymous mutations F67Y were involved in T200A. Conversely, the synonymous mutation 71S was triggered by the G213A SNP.

A significant difference was detected in the frequencies of all examined genes’ SNPs among pneumonic and healthy calves (*p* < 0.005). Chi-square analysis was carried out for comparison of the distribution of all identified SNPs in all genes between pneumonic and healthy calves. Total Chi-square value showed significant variation among the identified SNPs in all genes between resistant and affected animals (*p* < 0.05; Table 4).

Table 5 shows the discriminant analysis results for the arrangement of kinds of genes and healthy status. The organization consequences showed that the model properly classified 100% of the cases overall for both the CON group and the PG. These results indicate that the SNP markers included in the model possess a good level of discriminatory power and may be useful as potential genetic indicators for pneumonia susceptibility in calves.

### 3.4. Antioxidants and Immunological Profile

In comparison to the CON group, pneumonic calves showed a considerable drop in SOD, GSH, and TAC, and a large increase in MDA, a biomarker of oxidative stress (Table 5). A significant rise (*p* < 0.01) in serum concentrations of IL-1α (155.1 ± 3.9 pg/mL), IL-1β (141.3 ± 5.3 pg/mL), IL-6 (140.5 ± 1.2 pg/mL), TNF-α (117.1 ± 3.0 pg/mL), and IFN-γ (143.8 ± 8.2 pg/mL) was observed in pneumonic calves (PG) related to the CON group, indicating a strong innate immune response. In contrast, the anti-inflammatory cytokine IL-10 was significantly lesser (*p* < 0.01) in PG (27.5 ± 1.7 pg/mL) than in the CON group (Table 5).

### 3.5. Association Among Gene Expression Pattern and Serum Profile of Immunological and Antioxidant Biomarkers

Overall, gene expression pattern was strongly correlated with antioxidant or immunological biomarkers (Table 6).

## 4. Discussion

Due to its frequent occurrence, high rate of morbidity and mortality, slow growth, and veterinary costs, pneumonia is supposed to be a main cause of financial loss for calves of all ages and types [4]. This work aimed to monitor changes in gene expression and nucleotide sequences of immunological markers of pneumonic calves.

In comparison to the CON group, pneumonic calves demonstrated a marked increase in body temperature, respiratory rate, and pulse rate. Our clinical findings were comparable to that observed by [12,36,37,38,39,40].

We investigated the alterations in the immunological and antioxidant status of calves with pneumonia in comparison to healthy calves by assessing the mRNA values of immune and antioxidant biomarkers. The expression of the genes IL-1α, IL1B, IL6, TNF-α, IL10, and IFN-γ was much greater in the affected calves than in the CON group. This is the first report to thoroughly examine the transcription levels of the antioxidant and immunological biomarkers associated with pneumonia in calves. As a result, quantitative variations in the expression of the genes under investigation occur before pneumonia manifests itself. The immune genes’ mRNA levels in goats (SLC11A1, CD-14, CCL2, TLR1, TLR7, TLR8, TLR9, defensin, SP110, SPP1, BP1, A2M, ADORA3, CARD15, IRF3, and SCART1) varied between the healthy and infected goats with pneumonia [41]. Gene expression outlining revealed that Toll-like receptors (TLRs) and complement genes were accompanying infectious pneumonia in sheep [42]. When comparing pneumonia-affected sheep to resistant ewes, the IL-1α, IL1B, IL6, and TNF-α genes were markedly upregulated [43]. The gene that codes for IL10, on the other hand, was downregulated.

Our investigation used a PCR-DNA-sequencing procedure to illustrate the difference in immunological (IL-1α, IL1B, IL6, TNF-α, IL10, and IFN-γ) and antioxidant (PRDX6, ATG7, NDUFS6, and NOX4) genes in calves with pneumonia and healthy calves. The results demonstrate that there are transformations in the SNPs involving the two classes. It is fundamental to stress that, in contrast to the similar datasets obtained from GenBank, the polymorphisms discovered and presented, in this circumstance, offer extra evidence for the evaluated indicators. No research has looked at the relationship between calves’ risk of pneumonia and SNPs in the immune and antioxidant genes. This link is initially shown by the Bos taurus gene sequences used in our study, which were published in PubMed. However, ruminant susceptibility to pneumonia was assessed using the candidate gene technique. For instance, there is evidence linking MHC-DRB1 genotypes to either resistance or susceptibility to Mycoplasma ovipneumoniae in sheep [44]. Furthermore, it has been demonstrated that the consequences of ovine TMEM154 gene polymorphisms during normal exposure influence progressive pneumonia virus susceptibility [45]. There have been reports linking TLR gene variants to pneumococcal disease in humans [46]. Through PCR-DNA sequencing in Baladi goats with and without pneumonia, SNPs linked to pneumonia susceptibility and resistance were discovered in the immunological (SLC11A1, CD-14, CCL2, TLR1, TLR7, TLR8, TLR9, defensin, SP110, SPP1, BP1, A2M, ADORA3, CARD15, IRF3, and SCART1) genes [41].

Mutation is the central cause of selection and adaptability [47]. Exonic area mutations were presented in all examined markers under investigation in this case, leading to different coding DNA sequences in pneumonic as opposed to healthy calves. Eight non-synonymous and fourteen synonymous SNPs were identified through DNA sequencing of investigated genes. Protein sequences are altered by non-synonymous mutations, and natural selection usually targets animals with these changes [47]. Genetic discrepancy caused by non-synonymous SNPs adjusts the fixed amino acid at the mutant site, potentially leading to structural and practical deviations in the mutated protein [48]. It was long thought that there was either no selection on synonymous mutations or very little selection [47]. The examined immune genes must be precisely characterized at the molecular level in order to comprehend the physiological alterations between pneumonic and healthy calves in terms of resistance and susceptibility. According to our research, polymorphisms based on the translated DNA sequence of calves are further valued than intronic regions.

In the context of inflammation, cytokines, including NFKB, IL-1α, TNF-α, IFN-γ, IL-6, and IL1B, act as indirect biomarkers [49]. IL-10 is an anti-inflammatory cytokine that prevents macrophages from producing natural killer cells (IL-1 and TNF) and Th1 lymphocytes from producing IFN- and IL-2 [50,51]. Through its interaction with regulating suppressor T CD8+ cells, IL-10, which is formed by a variety of T cells, has been shown to contain anti-inflammatory characteristics that defend uterine tissues from the highly pathogenic impact of inflammatory cells and mediators [52]. The IL10, TNF-α, IL1B, and IL6 genes were PCR-DNA sequenced to identify SNPs with nucleotide sequence variations connected to diarrhea resistance or vulnerability in lambs [53]. Diarrheal lambs had noticeably greater intensities of TNF-α, IL1B, and IL6 gene expression than resistant lambs. In diarrheal lambs, the IL10 gene was significantly downregulated compared to resistant lambs. The nucleotide sequences of healthy and endometritis-affected cows were found to differ by using PCR-DNA sequencing for the IL10 gene. The IL10 gene was expressed at much lower levels in cows suffering from endometritis [54].

A conserved ionized thiol group enables the peroxiredoxin (PRDX) family of antioxidant oxidoreductase enzymes to induce the reduction of hydrogen peroxide (H_2_O_2_). Thiol-specific peroxidases act as sensors for signaling events triggered by hydrogen peroxide and contribute to cellular defense, contrary to oxidative stress, by detoxifying peroxides and sulfur-containing radicals [55]. Complex I, the major enzyme complex in the mitochondrial electron transport chain, is encoded in part by the NADH ubiquinone oxidoreductase subunit S6 (NDUFS6) gene [56]. Through the action of Complex I, NADH donates electrons to the mitochondrial respiratory chain. Mutations in the NDUFS6 gene can lead to mitochondrial Complex I deficiency, which is associated with neurodegenerative diseases in adults, as well as severe illness in neonates [56]. It has formerly been stated that the NDUFS6 gene, set on BTA20 in cattle, has a 900 bp deletion that removes exon 2 entirely [57]. There is a measurable trait locus for SCS in this area of the genome [58].

PCR-DNA sequence revealed nucleotide sequence differences in the PRDX6 and NDUFS6 genes among control and mastitis-affected she-camels [59]. Significantly lower levels of PRDX6 and NDUFS6 gene expression were observed in mastitic camels. Differences in the PRDX6 gene’s nucleotide sequence were observed between calves with diarrhea and healthy calves [60]. Diarrheic calves have significantly higher levels of PRDX6 expression than resistant calves. In buffaloes with endometritis, NDUFS6 was expressed at significantly lower levels [61].

Numerous autophagy-associated genes (ATGs) are implicated in molecular pathways, and autophagy is a complicated and highly controlled process [62]. According to [63], ATG7 is an E1-like ligase that facilitates the elongation of separation membranes and is essential for the development of autophagosomes. According to [64], the expression of important genes linked to antioxidant replies, such as the ATG7 gene, was markedly downregulated by bacterial LPS. The catalytic NOX subunit of NADPH oxidase 4 (NOX4), a member of the NADPH oxidase family, converts electrons from NADPH to oxygen to produce ROS [65]. Numerous pathogeneses, such as cell senescence [66], apoptosis [67], endothelial dysfunction [68], angiogenesis [69], atherosclerosis and vascular ageing [70], cardiac remodeling [71], and neoplasms [72], have been linked to the NOX4 gene. NOX4 gene polymorphism alters the relationships between dietary caloric consumption and peripheral blood mononuclear cell ROS levels [73].

The altered immune expression patterns observed in calves with pneumonia may result from the induction of inflammatory and antioxidant biomarkers triggered by lipopolysaccharides or endotoxins [74]. When LPSs excite macrophages, inflammatory gene expressions treble, mRNA levels increase by approximately one-hundred-fold, and the amount of protein released can rise by up to 10,000 times [75]. Moreover, damaged tissues generate more free radical reactions than healthy tissues [76,77]. In contrast to the resistant genotypes, which considerably grow the expressions of IL-2 and IL-10 to suppress the inflammatory response, the vulnerable genotypes increase the inflammatory response via the large release of inflammatory biomarkers [78]. Calves with pneumonia also produce a range of cellular immunological mechanisms that bind to cell surface receptors to arbitrate and control the inflammatory response and immune activity [79]. Numerous investigations have shown that different cell immunological components have intricate interactions. The formation of various immune globulins, complements, and acute phase reactive proteins, for example, has been discovered to be influenced by the interactions of these proteins, resulting in a complex network structure [80]. We therefore expect that the majority of the pneumonia cases in our analysis were caused by infectious pneumonia. Moreover, our real-time PCR results give a credible suggestion that animals suffering from pneumonia have a robust inflammatory reaction.

The obvious drop in the values of RBCs, hemoglobin, and PCV (%) in pneumonic calves is accordant with conclusions of other works [20,37,81]. Pneumonic calves showed a significant reduction in lymphocytes but a considerable rise in WBCs and neutrophils when compared to the CON group. These findings were compatible with those of [20,37,81,82]. Increases in WBC counts, particularly neutrophils, are frequently observed in a variety of disorders as a result of inflammatory courses, like those in respiratory disorders. According to [83,84], inflammatory lesions and the attendance of bacterial infection may be the cause of the significant increase in WBCs and neutrophils that happened in pneumonia. According to [82,85], calves with respiratory infections may experience severe lymphopenia as a result of the high-concentration endotoxin’s ability to trigger lymphocyte lysis. However, the significant reduction in lymphocytes may be due to immunological suppression brought on by a variety of stressors or may be explained by the adrenal gland being stimulated by tissue infected by bacterial toxins [86,87].

When oxidants and antioxidants are not balanced, oxidative stress occurs, which damages cells and tissues. Oxidative stress eventually results in mechanical injury and loss of function in cells and tissues when there is an imbalance caused by an increase in oxidants or a decrease in antioxidants. This can have a wide range of detrimental effects on animals, from decreased production to death. Thus, it has been reported that oxidative stress had a significant purpose in the etiology and pathogenesis of a number of animal diseases, particularly those affecting the respiratory system [88]. Calves with pneumonia had significantly higher MDA values and significantly lower SOD, GSH, and TAC activity in their oxidant/antioxidant profiles as opposed to the CON group. Our findings were close to that observed by [40,89,90,91]. By inhibiting the peroxidation procedure and the generation of additional free radical molecules [92], the rise in serum MDA was linked to cellular lipid peroxidation, and the fall in SOD, GSH, and TAC levels was linked to their consumption in cells’ defense against oxidative damage.

According to this investigation, PG showed a significant rise in pro-inflammatory cytokines in contrast to the CON group. Our findings were comparable to that found by [20,36,40] in cattle calves [93], in buffalo calves [94], in camel calves [95,96,97], in sheep [37], and in cattle and in pigs [98]. As per [95], reduced anti-inflammatory cytokine levels in the pneumonic group likely exacerbated the illness by facilitating an aggressive innate immune response. The inflammatory response during infection is made worse by this misalignment of pro- and anti-inflammatory signals [97]. In the early stages of bacterial infection, resident alveolar macrophages are stimulated and release pro-inflammatory cytokines, eventually leading to the formation of a pro-inflammatory amplification loop between lymphocytes and local or recruited neutrophils or macrophages. A repeat recurrence of anti-inflammatory cytokines is generated as the infection is eradicated in order to pinpoint the location of the inflammatory response in the lung microenvironment and, eventually, to suppress this immune response [99].

This current work has several restrictions that should be addressed in upcoming research. First, this study focused on a single breed, so applying this research to different calf breeds would provide more accurate health assessments. Second, only a limited set of immunity and antioxidant-related genes were examined, indicating the need for broader gene profiling in subsequent studies. Third, bacterial species and viruses present in the pneumonic calves were not isolated or identified, and future research should aim to address them. Finally, histological alterations in the infected lungs were not evaluated in this study; thus, analyzing the histopathological changes is essential for a comprehensive understanding of the disease.

## 5. Conclusions

Our results emphasize the nucleotide sequence variations and the expression profiles of the immunological and antioxidant genes studied as a cause or a mechanism-mediated response for pneumonia resistance or susceptibility in calves. The findings provide strong evidence of noteworthy immunological and oxidative fluctuations associated with pneumonia in calves, particularly concerning their blood antioxidant and immunological profiles. The distinct expression patterns of pneumonia-resistant and susceptible calves may represent biomarkers and references to monitor these animals’ health. In line with genetic markers interrelated to innate immunity to infection, these results present a viable method for lowering calves’ risk of pneumonia through discriminatory breeding.

## Figures and Tables

**Table 1:** Forward and reverse oligonucleotide primers for real-time PCR targeting the immunological and antioxidant genes under investigation.

Examined Indicator	Primer	Product Size (bp)	Annealing Temperature (°C)	GenBank Isolate
*IL-1α*	F5′-GTCCCTGACCTCTTTGAAGACCT-3 R5′-ACGTTACTCTGGAAGCTGTAATG-3′	356	60	NM_174092.1
*IL-1β*	F5′-GAGAATGAGCTGTTATTTGAG-3 R5′-GCTCATGCAGAACACCACTTC-3′	423	58	NM_174093.1
*IL-6*	F5′-AGCGCCTTCACTCCATTCGCT-3′ R5′-CTCATACTCGTTCTGGAGGTAG-3′	384	58	NM_173923.2
*IL10*	F5′-TACCTGGGTTGCCAAGCCTTGTC-3′ R5′-CTTTTGCATCTTCGTTGTCAT-3′	306	58	NM_174088.1
*TNFα*	F5′-TCCTTCCTCCTGGTTGCAGGAGC-3′ R5′-CTCCTCCCTGGTAGATGGGTTC-3′	490	60	NM_173966.3
*IFN-γ*	F5′-TATACAAGCTATTTCTTAGCT-3′ R5′-AGAGCTGCCATTCAAGAACTTC-3′	321	58	NM_174086.1
*PRDX6*	F5′-ACGGAGCTCGGCAGAGCAGCA-3′ R5′-ATGGTTGGAAGGACCATCACGC-3′	434	60	NM_174643.1
*ATG7*	F5′-GGTGTCAGACACATCACGTTCGT-3′ R5′-CTGGGCTTCTTCAGGCCGTGTC-3′	416	60	NM_001142967.1
*NDUFS6*	F5′-ACGCGCGGCCTGCCCGGGGGC-3′ R5′-TCCTGTCCTGGAAGCTCTGTGCG-3′	405	58	XM_005221658.4
*NOX4*	F5′-CACCTCTGCCTGCTTATCTGGC-3′ R5′-TCGGTATCTTGCTGCATTCAGT-3′	396	60	NM_001304775.1
*GAPDH*	F5′-CCTGCCCGTTCGACAGATAG-3′ R5′-ATGGCGACGATGTCCACTTT-3′	153	58	NM_001034034.2

IL1α, interleukin-1 alpha; IL1β, interleukin-1beta; IL6, interleukin 6; IL10, interleukin 10; TNFα, tumor necrosis factor alpha; INF_γ_, interferon gamma; PRDX6, peroxiredoxin 6; AT7G, autophagy-related gene 7; NDUFS6, NADH/ubiquinone oxidoreductase subunit S6; NOX4, NADPH oxidase 4; GAPDH, Glyceraldehyde-3-Phosphate Dehydrogenase.

**Table 2:** Clinical examination of healthy and pneumonic calves.

Parameters	Healthy Calves	BRD Calves	*p*-Values
Temperature (°C)	38.8 ± 0.05	41.3 ± 0.3 *	0.005
Pulse rate (beat/min)	93.8 ± 0.4	145.4 ± 4.5 *	0.002
Respiration rate (breath/min)	29.4 ± 0.3	51.9 ± 2.3 *	0.003

Significant changes among the two groups shown as *: *p* < 0.05.

**Table 3:** Hematological parameters of apparently health and pneumonic calves.

Parameters	Healthy Calves	Pneumonic Calves	*p*-Values
RBCs (×10^6^/µL)	6.3 ± 0.6	4.2 ± 0.5 *	0.001
Hb (g/dL)	11.9 ± 1.5	8.1 ± 1.01 *	0.001
PCV (%)	32.1 ± 0.5	22 ± 0.3 *	0.001
MCV (fl)	34.4 ± 1.2	32.9 ± 0.5	0.1
MCH (pg)	22.1 ± 1.9	19.1 ± 0.88	0.09
MCHC (%)	36.1 ± 1.4	33.4 ± 0.8	0.07
WBC (×10^3^/µL)	11.22 ± 1.4	18.11 ± 3.32 *	0.001
Neutrophils (×10^3^/µL)	4.02 ± 0.08	8.99 ± 0.29 *	0.004
Lymphocyte (×10^3^/µL)	4.1 ± 0.08	2.11 ± 0.55 *	0.03

WBC, total leukocytes count; RBCs, erythrocytes count; Hb, hemoglobin; PCV, packed cell volume; MCV, mean corpuscular volume; MCH, mean corpuscular hemoglobin; MCHC, mean corpuscular hemoglobin concentration. Significant differences between the two groups are indicated by *: *p* < 0.05.

**Table 4:** Pneumonic and healthy calves’ immunological and antioxidant biomarker distributions with a single base differential and possible genetic change.

Genes	SNPs	Healthy *n* = 45	Pneumonia *n* = 180	Total *n* = 225	kind of Inherited Change	Amino Acid Order and Sort	Chi Squar Value X^2^	*p*-Value
*IL-1α*	G56C	21/45	-/180	21/225	Non-synonymous	19 S to R	92.6	<0.001
	A182G	34/45	-/180	34/225	Non-synonymous	61 K to R	149.2	<0.001
	T300C	-/45	67/180	67/225	Synonymous	100 N	23.8	<0.001
*IL-1β*	C153T	19/45	-/180	18/225	Synonymous	51 I	37.5	<0.001
*IL-6*	G221A	-/45	131/180	131/225	Non-synonymous	74 S to N	78.3	<0.001
	G323A	28/45	-/180	28/225	Non-synonymous	108 R to K	127.9	<0.001
*IL-10*	A105T	39/45	-/180	39/225	Synonymous	35 S	188.7	<0.001
	G129C	-/45	51/180	51/225	Synonymous	43 L	16.4	<0.001
	C168T	-/45	76/180	76/225	Synonymous	56 C	28.6	<0.001
	G203A	37/45	-/180	37/225	Non-synonymous	68 R to K	177.1	<0.001
*TNFα*	A120G	26/45	-/180	26/225	Synonymous	40 Q	117.5	<0.001
	C213T	-/45	39/180	39/225	Synonymous	71 D	11.7	<0.001
	T468C	18/45	-/180	18/225	Synonymous	156 Y	78.2	<0.001
*IFN-γ*	G35T	-/45	58/180	58/225	Non-synonymous	12 G to V	19.5	<0.001
*PRDX6*	T117C	-/45	82/180	82/225	Synonymous	39 N	20.4	<0.001
	T225C	-/45	126/180	126/225	Synonymous	75 D	71.5	<0.001
*AT7G*	T48C	38/45	-/180	38/225	Synonymous	16 S	182.8	<0.001
	G214A	31/45	-/180	31/225	Non-synonymous	72 V to I	143.8	<0.001
	C333T	19/45	-/180	19/225	Synonymous	111 A	83	<0.001
*NDUFS6*	G354A	-/45	103/180	103/180	Synonymous	118 S	47.9	<0.001
*NOX4*	T200A	28/45	-/180	28/225	Non-synonymous	67 F to Y	127.9	<0.001
	G213A	21/45	-/180	21/225	Synonymous	71 S	92.6	<0.001

IL1α, interleukin-1 alpha; IL1β, interleukin-1beta; IL6, interleukin 6; IL10, interleukin 10; TNFα, tumor necrosis factor alpha; INF_γ_, interferon gamma; PRDX6, peroxiredoxin 6; AT7G, autophagy-related gene 7; NDUFS6, NADH/ubiquinone oxidoreductase subunit S6; NOX4, NADPH oxidase 4; A = alanine; C = cysteine; D = aspartic acid; F = phenylalanine; G = glycine; I = isoleucine; K = lysine; L = leucine; N = asparagine; Q = glutamine; R = arginine; S = serine; V = valine; and Y = tyrosine.

**Table 5:** Antioxidants and immunological parameters of control and pneumonic calves.

Parameters	Healthy Calves	BRD Calves	*p*-Values
SOD (U/mL)	55 ± 0.5	40 ± 0.5 *	0.001
GSH (mg/dl)	37.6 ± 0.8	26.3 ± 0.8 *	0.001
TAC (mM/L)	49 ± 0.5	35 ± 0.5 *	0.001
MDA (nmol/mL)	5.8 ± 0.05	10 ± 0.1 *	0.002
IL1α (pg/mL)	32.6 ± 2.7	155.1 ± 3.9 *	0.002
IL1β (pg/mL)	25.4 ± 1.6	141.3 ± 5.3 *	0.001
IL 6 (pg/mL)	26 ± 1.2	140.5 ± 1.2 *	0.001
IL 10 (pg/mL)	119.4 ± 2.5	27.5 ± 1.7 *	0.002
TNFα (pg/mL)	29.3 ± 1.9	117.1 ± 3 *	0.001
IFN_γ_ (Pg/mL)	46.7 ± 0.9	143.8 ± 8.2 *	0.007

SOD, superoxide dismutase; GSH, glutathione; TAC, total antioxidant capacity; MDA, malondialdehyde; IL1α, interleukin-1 alpha; IL1β, interleukin-1beta; IL6, interleukin 6; IL10, interleukin 10; TNFα, tumor necrosis factor alpha; INF_γ_, interferon gamma. *: *p* < 0.05

**Table 6:** Correlation between gene expression pattern and serum profile of immunological biomarkers.

Gene Expression	Serum Profile
PRDX6	SOD (r = 0.877 and *p* = 0.024) GSH (r = 0.924 and *p* = 0.008) TAC (r = 0.961 and *p* = 0.0.002)
NDUFS6	SOD (r = 0.917 and *p* = 0.01) GSH (r = 0.925 and *p* = 0.008) TAC (r = 0.904 and *p* = 0.0.01) MDA (r = −0.887 and *p* = 0.01)
NOX4	SOD (r = −0.975 and *p* = 0.001) GSH (r = −0.973 and *p* = 0.001) TAC (r = −0.9634 and *p* = 0.003) MDA (r = 0.951 and *p* = 0.003)
ATG7	GSH (r = 0.853 and *p* = 0.013) TAC (r = 0.849 and *p* = 0.03)
IL1α	INF_γ_ (r = −0.997 and *p* = 0.04)
IL10	TNFα (r = −0.998 and *p* = 0.04)
INF_γ_	TNFα (r = 1 and *p* = 0.005)

PRDX6, peroxiredoxin 6; NDUFS6, NADH/ubiquinone oxidoreductase subunit S6; NOX4, NADPH oxidase 4; AT7G, autophagy-related gene 7; IL1α, interleukin-1 alpha; IL10, interleukin 10; INF_γ_, interferon gamma.

## Data Availability

The relevant author will grant supporting material for the study’s results upon reasonable request.

## Supplementary Materials

The following supporting information can be downloaded at https://www.mdpi.com/article/10.3390/vetsci12070620/s1, Figure S1: An example of the alignment of the IL-1α gene (356 bp) between calves with pneumonia (P) and that are healthy (H). Figure S2: An example of the alignment of the IL-1β gene (423 bp) among calves with pneumonia (P) and that are healthy (H). Figure S3: An example of the alignment of the IL-6 gene (384 bp) concerning calves with pneumonia (P) and that are healthy (H). Figure S4: An example of the alignment of the IL-10 gene (306 bp) amongst calves with pneumonia (P) and that are healthy (H). Figure S5: An example of the alignment of the TNFα gene (490-bp) between calves with pneumonia (P) and that are healthy (H). Figure S6: An example of the alignment of the IFN-γ gene (321-bp) between calves with pneumonia (P) and that are healthy (H). Figure S7: An example of the alignment of the PRDX6 gene (434-bp) between calves with pneumonia (P) and that are healthy (H). Figure S8: An example of the alignment of the ATG7 gene (416-bp) between calves with pneumonia (P) and that are healthy (H). Figure S9: An example of the alignment of the NDUFS6 gene (405-bp) between calves with pneumonia (P) and that are healthy (H). Figure S10: An example of the alignment of the NOX4 gene (396-bp) between calves with pneumonia (P) and that are healthy (H).
