# Supplementary material for: Variants in Nucleotide Sequences; Gene Expression; and Hematological, Immune, and Antioxidant Biomarkers Linked to Pneumonia Risk in Holstein Calves
Source: Vet Sci. 2025 Jun 26;12(7):620. doi: 10.3390/vetsci12070620 (PMC12300230; doi:10.3390/vetsci12070620)
Supplement: Supplementary file 1 [file vetsci-12-00620-s001.zip › vetsci-3621061-supplementary.pdf]

```
NM_174092.1      GTCCCTGACCTCTTTGAAGACCTGAAGAACTGTTACAGTGAAAATGAAGACTACAGTTCT      60
H                GTCCCTGACCTCTTTGAAGACCTGAAGAACTGTTACAGTGAAAATGAAGACTACACTTCT      60
P                GTCCCTGACCTCTTTGAAGACCTGAAGAACTGTTACAGTGAAAATGAAGACTACAGTTCT      60
                 ******************************************************** ****

NM_174092.1      GAAATTGACCACCTCTCTCTCAATCAGAAGTCCTTCTATGATGCAAGCTATGAGCCACTT      120
H                GAAATTGACCACCTCTCTCTCAATCAGAAGTCCTTCTATGATGCAAGCTATGAGCCACTT      120
P                GAAATTGACCACCTCTCTCTCAATCAGAAGTCCTTCTATGATGCAAGCTATGAGCCACTT      120
                 ************************************************************

NM_174092.1      CGTGAGGACCAGATGAATAAGTTTATGTCCCTGGATACCTCGGAAACCTCTAAGACATCC      180
H                CGTGAGGACCAGATGAATAAGTTTATGTCCCTGGATACCTCGGAAACCTCTAAGACATCC      180
P                CGTGAGGACCAGATGAATAAGTTTATGTCCCTGGATACCTCGGAAACCTCTAAGACATCC      180
                 ************************************************************

NM_174092.1      AAGCTTAGCTTCAAGGAGAATGTGGTGATGGTGGCAGCCAGTGGGAAGATTCTGAAGAAG      240
H                AGGCTTAGCTTCAAGGAGAATGTGGTGATGGTGGCAGCCAGTGGGAAGATTCTGAAGAAG      240
P                AAGCTTAGCTTCAAGGAGAATGTGGTGATGGTGGCAGCCAGTGGGAAGATTCTGAAGAAG      240
                 * **********************************************************

NM_174092.1      AGACGGTTGAGTTTAAATCAGTTCATCACCGATGATGACCTGGAAGCCATTGCCAATAAT      300
H                AGACGGTTGAGTTTAAATCAGTTCATCACCGATGATGACCTGGAAGCCATTGCCAATAAT      300
P                AGACGGTTGAGTTTAAATCAGTTCATCACCGATGATGACCTGGAAGCCATTGCCAATAAC      300
                 ***********************************************************

NM_174092.1      ACAGAAGAAGAAATCATCAAGCCCAGATCAGCACATTACAGCTTCCAGAGTAACGT 356
H                ACAGAAGAAGAAATCATCAAGCCCAGATCAGCACATTACAGCTTCCAGAGTAACGT 356
P                ACAGAAGAAGAAATCATCAAGCCCAGATCAGCACATTACAGCTTCCAGAGTAACGT 356
                 ********************************************************
```

Figure S1: An example of the alignment of the *IL-1α* gene (356 bp) between calves with pneumonia (P) and healthy (H).

```
NM_174093.1      GAGAATGAGCTGTTATTTGAGGCTGATGACCCTAAACAGATGAAGAGCTGCATCCAACAC      60
H                GAGAATGAGCTGTTATTTGAGGCTGATGACCCTAAACAGATGAAGAGCTGCATCCAACAC      60
P                GAGAATGAGCTGTTATTTGAGGCTGATGACCCTAAACAGATGAAGAGCTGCATCCAACAC      60
                 ************************************************************

NM_174093.1      CTGGACCTCGGTTCCATGGGAGATGGAAACATCCAGCTGCAGATTTCTCACCAGTTCTAC      120
H                CTGGACCTCGGTTCCATGGGAGATGGAAACATCCAGCTGCAGATTTCTCACCAGTTCTAC      120
P                CTGGACCTCGGTTCCATGGGAGATGGAAACATCCAGCTGCAGATTTCTCACCAGTTCTAC      120
                 ************************************************************

NM_174093.1      AACAAAAGCTTCAGGCAGGTGGTGTCGGTCATCGTGGCCATGGAGAAGCTGAGGAACAGT      180
H                AACAAAAGCTTCAGGCAGGTGGTGTCGGTCATTGTGGCCATGGAGAAGCTGAGGAACAGT      180
P                AACAAAAGCTTCAGGCAGGTGGTGTCGGTCATCGTGGCCATGGAGAAGCTGAGGAACAGT      180
                 ******************************** ***************************

NM_174093.1      GCCTACGCACATGTCTTCCATGATGATGACCTGAGGAGCATCCTTTCATTCATCTTTGAA      240
H                GCCTACGCACATGTCTTCCATGATGATGACCTGAGGAGCATCCTTTCATTCATCTTTGAA      240
P                GCCTACGCACATGTCTTCCATGATGATGACCTGAGGAGCATCCTTTCATTCATCTTTGAA      240
                 ************************************************************

NM_174093.1      GAAGAGCCTGTCATCTTCGAAACGTCCTCCGACGAGTTTCTGTGTGACGCACCCGTGCAG      300
H                GAAGAGCCTGTCATCTTCGAAACGTCCTCCGACGAGTTTCTGTGTGACGCACCCGTGCAG      300
P                GAAGAGCCTGTCATCTTCGAAACGTCCTCCGACGAGTTTCTGTGTGACGCACCCGTGCAG      300
                 ************************************************************

NM_174093.1      TCAATAAAGTGCAAACTCCAGGACAGAGAGCAAAAATCCCTGGTGCTGGCTAGCCCATGT      360
H                TCAATAAAGTGCAAACTCCAGGACAGAGAGCAAAAATCCCTGGTGCTGGCTAGCCCATGT      360
P                TCAATAAAGTGCAAACTCCAGGACAGAGAGCAAAAATCCCTGGTGCTGGCTAGCCCATGT      360
                 ************************************************************

NM_174093.1      GTGCTGAAGGCTCTCCACCTCCTCTCACAGGAAATGAACCGAGAAGTGGTGTTCTGCATG      420
H                GTGCTGAAGGCTCTCCACCTCCTCTCACAGGAAATGAACCGAGAAGTGGTGTTCTGCATG      420
P                GTGCTGAAGGCTCTCCACCTCCTCTCACAGGAAATGAACCGAGAAGTGGTGTTCTGCATG      420
                 ************************************************************

NM_174093.1      AGC        423
H                AGC        423
P                AGC        423
                 ***
```

Figure S2: An example of the alignment of the *IL-1β* gene (423 bp) between calves with pneumonia (P) and healthy (H).

```
NM_173923.2     AGCGCCTTCACTCCATTCGCTGTCTCCCTGGGGCTGCTCCTGGTGATGACTTCTGCTTTC     60
H               AGCGCCTTCACTCCATTCGCTGTCTCCCTGGGGCTGCTCCTGGTGATGACTTCTGCTTTC     60
P               AGCGCCTTCACTCCATTCGCTGTCTCCCTGGGGCTGCTCCTGGTGATGACTTCTGCTTTC     60
                ************************************************************

NM_173923.2     CCTACCCCGGGTCCCCTGGGAGAAGATTTCAAAAATGACACCACCCCAGGCAGACTACTT     120
H               CCTACCCCGGGTCCCCTGGGAGAAGATTTCAAAAATGACACCACCCCAGGCAGACTACTT     120
P               CCTACCCCGGGTCCCCTGGGAGAAGATTTCAAAAATGACACCACCCCAGGCAGACTACTT     120
                ************************************************************

NM_173923.2     CTGACCACTCCAGAGAAAACCGAAGCTCTCATTAAGCGCATGGTCGACAAAATCTCTGCA     180
H               CTGACCACTCCAGAGAAAACCGAAGCTCTCATTAAGCGCATGGTCGACAAAATCTCTGCA     180
P               CTGACCACTCCAGAGAAAACCGAAGCTCTCATTAAGCGCATGGTCGACAAAATCTCTGCA     180
                ************************************************************

NM_173923.2     ATGAGAAAGGAGATATGTGAGAAGAATGATGAGTGTGAAAGCAGCAAGGAGACACTGGCA     240
H               ATGAGAAAGGAGATATGTGAGAAGAATGATGAGTGTGAAAGCAGCAAGGAGACACTGGCA     240
P               ATGAGAAAGGAGATATGTGAGAAGAATGATGAGTGTGAAAACAGCAAGGAGACACTGGCA     240
                **************************************** *******************

NM_173923.2     GAAAATAAGCTGAATCTTCCAAAAATGGAGGAAAAGGACGGATGCTTCCAATCTGGGTTC     300
H               GAAAATAAGCTGAATCTTCCAAAAATGGAGGAAAAGGACGGATGCTTCCAATCTGGGTTC     300
P               GAAAATAAGCTGAATCTTCCAAAAATGGAGGAAAAGGACGGATGCTTCCAATCTGGGTTC     300
                ************************************************************

NM_173923.2     AATCAGGCGATTTGCTTGATCAGAACCACTGCTGGTCTTCTGGAGTATCAGATATACCTG     360
H               AATCAGGCGATTTGCTTGATCAAAACCACTGCTGGTCTTCTGGAGTATCAGATATACCTG     360
P               AATCAGGCGATTTGCTTGATCAGAACCACTGCTGGTCTTCTGGAGTATCAGATATACCTG     360
                ********************** *************************************

NM_173923.2     GACTACCTCCAGAACGAGTATGAG     384
H               GACTACCTCCAGAACGAGTATGAG     384
P               GACTACCTCCAGAACGAGTATGAG     384
                ************************
```

Figure S3: An example of the alignment of the *IL-6* gene (384 bp) between calves with pneumonia (P) and healthy (H).

```
NM_174088.1      TACCTGGGTTGCCAAGCCTTGTCGGAAATGATCCAGTTTTACCTGGAAGAGGTGATGCCA      60
H                TACCTGGGTTGCCAAGCCTTGTCGGAAATGATCCAGTTTTACCTGGAAGAGGTGATGCCA      60
P                TACCTGGGTTGCCAAGCCTTGTCGGAAATGATCCAGTTTTACCTGGAAGAGGTGATGCCA      60
                 ************************************************************

NM_174088.1      CAGGCTGAGAACCACGGGCCTGACATCAAGGAGCACGTGAACTCACTGGGGGAGAAGCTG      120
H                CAGGCTGAGAACCACGGGCCTGACATCAAGGAGCACGTGAACTCTCTGGGGGAGAAGCTG      120
P                CAGGCTGAGAACCACGGGCCTGACATCAAGGAGCACGTGAACTCACTGGGGGAGAAGCTG      120
                 ******************************************** ***************

NM_174088.1      AAGACCCTGCGGCTGCGGCTGCGGCGCTGTCATCGCTTTCTGCCCTGCGAAAACAAGAGC      180
H                AAGACCCTGCGGCTGCGGCTGCGGCGCTGTCATCGCTTTCTGCCCTGCGAAAACAAGAGC      180
P                AAGACCCTCCGGCTGCGGCTGCGGCGCTGTCATCGCTTTCTGCCCTGTGAAAACAAGAGC      180
                 ******** ************************************** ************

NM_174088.1      AAGGCGGTGGAGAAGGTGAAGAGAGTCTTCAGTGAGCTCCAAGAGAGGGGTGTCTACAAA      240
H                AAGGCGGTGGAGAAGGTGAAGAAAGTCTTCAGTGAGCTCCAAGAGAGGGGTGTCTACAAA      240
P                AAGGCGGTGGAGAAGGTGAAGAGAGTCTTCAGTGAGCTCCAAGAGAGGGGTGTCTACAAA      240
                 ********************** *************************************

NM_174088.1      GCCATGAGTGAGTTTGACATCTTCATCAACTACATAGAAACCTACATGACAACGAAGATG      300
H                GCCATGAGTGAGTTTGACATCTTCATCAACTACATAGAAACCTACATGACAACGAAGATG      300
P                GCCATGAGTGAGTTTGACATCTTCATCAACTACATAGAAACCTACATGACAACGAAGATG      300
                 ************************************************************

NM_174088.1      CAAAAG    306
H                CAAAAG    306
P                CAAAAG    306
                 ******
```

Figure S4: An example of the alignment of the *IL-10* gene (306 bp) between calves with pneumonia (P) and healthy (H).

```
NM_173966.3      TCCTTCCTCCTGGTTGCAGGAGCCACCACGCTCTTCTGCCTGCTGCACTTCGGGGTAATC      60
H                TCCTTCCTCCTGGTTGCAGGAGCCACCACGCTCTTCTGCCTGCTGCACTTCGGGGTAATC      60
P                TCCTTCCTCCTGGTTGCAGGAGCCACCACGCTCTTCTGCCTGCTGCACTTCGGGGTAATC      60
                 ************************************************************

NM_173966.3      GGCCCCCAGAGGGAAGAGCAGTCCCCAGGTGGCCCCTCCATCAACAGCCCTCTGGTTCAA      120
H                GGCCCCCAGAGGGAAGAGCAGTCCCCAGGTGGCCCCTCCATCAACAGCCCTCTGGTTCAG      120
P                GGCCCCCAGAGGGAAGAGCAGTCCCCAGGTGGCCCCTCCATCAACAGCCCTCTGGTTCAA      120
                 ***********************************************************

NM_173966.3      ACACTCAGGTCCTCTTCTCAAGCCTCAAGTAACAAGCCGGTAGCCCACGTTGTAGCCGAC      180
H                ACACTCAGGTCCTCTTCTCAAGCCTCAAGTAACAAGCCGGTAGCCCACGTTGTAGCCGAC      180
P                ACACTCAGGTCCTCTTCTCAAGCCTCAAGTAACAAGCCGGTAGCCCACGTTGTAGCCGAC      180
                 ************************************************************

NM_173966.3      ATCAACTCTCCGGGGCAGCTCCGGTGGTGGGACTCGTATGCCAATGCCCTCATGGCCAAC      240
H                ATCAACTCTCCGGGGCAGCTCCGGTGGTGGGACTCGTATGCCAATGCCCTCATGGCCAAC      240
P                ATCAACTCTCCGGGGCAGCTCCGGTGGTGGGATTCGTATGCCAATGCCCTCATGGCCAAC      240
                 ******************************** ***************************

NM_173966.3      GGTGTGAAGCTGGAAGACAACCAGCTGGTGGTGCCTGCTGACGGGCTTTACCTCATCTAC      300
H                GGTGTGAAGCTGGAAGACAACCAGCTGGTGGTGCCTGCTGACGGGCTTTACCTCATCTAC      300
P                GGTGTGAAGCTGGAAGACAACCAGCTGGTGGTGCCTGCTGACGGGCTTTACCTCATCTAC      300
                 ************************************************************

NM_173966.3      TCACAGGTCCTCTTCAGGGGCCAAGGCTGCCCTTCCACCCCCTTGTTCCTCACCCACACC      360
H                TCACAGGTCCTCTTCAGGGGCCAAGGCTGCCCTTCCACCCCCTTGTTCCTCACCCACACC      360
P                TCACAGGTCCTCTTCAGGGGCCAAGGCTGCCCTTCCACCCCCTTGTTCCTCACCCACACC      360
                 ************************************************************

NM_173966.3      ATCAGCCGCATTGCAGTCTCCTACCAGACCAAGGTCAACATCCTGTCTGCCATCAAGAGC      420
H                ATCAGCCGCATTGCAGTCTCCTACCAGACCAAGGTCAACATCCTGTCTGCCATCAAGAGC      420
P                ATCAGCCGCATTGCAGTCTCCTACCAGACCAAGGTCAACATCCTGTCTGCCATCAAGAGC      420
                 ************************************************************

NM_173966.3      CCTTGCCACAGGGAGACCCCAGAGTGGGCTGAGGCCAAGCCCTGGTATGAACCCATCTAC      480
H                CCTTGCCACAGGGAGACCCCAGAGTGGGCTGAGGCCAAGCCCTGGTACGAACCCATCTAC      480
P                CCTTGCCACAGGGAGACCCCAGAGTGGGCTGAGGCCAAGCCCTGGTATGAACCCATCTAC      480
                 *********************************************** ************

NM_173966.3      CAGGGAGGAG 490
H                CAGGGAGGAG 490
P                CAGGGAGGAG 490
                 **********
```

Figure S5: An example of the alignment of the *TNFα* gene (490-bp) between between calves with pneumonia (P) and healthy (H).

```
NM_174086.1      TATACAAGCTATTTCTTAGCTTTACTGCTCTGTGGGCTTTTGGGTTTTTCTGGTTCTTAT      60
H                TATACAAGCTATTTCTTAGCTTTACTGCTCTGTGGGCTTTTGGGTTTTTCTGGTTCTTAT      60
P                TATACAAGCTATTTCTTAGCTTTACTGCTCTGTGTGCTTTTGGGTTTTTCTGGTTCTTAT      60
                 ********************************** *************************

NM_174086.1      GGCCAGGGCCAATTTTTTAGAGAAATAGAAAACTTAAAGGAGTATTTTAATGCAAGTAGC      120
H                GGCCAGGGCCAATTTTTTAGAGAAATAGAAAACTTAAAGGAGTATTTTAATGCAAGTAGC      120
P                GGCCAGGGCCAATTTTTTAGAGAAATAGAAAACTTAAAGGAGTATTTTAATGCAAGTAGC      120
                 ************************************************************

NM_174086.1      CCAGATGTAGCTAAGGGTGGGCCTCTCTTCTCAGAAATTTTGAAGAATTGGAAAGATGAA      180
H                CCAGATGTAGCTAAGGGTGGGCCTCTCTTCTCAGAAATTTTGAAGAATTGGAAAGATGAA      180
P                CCAGATGTAGCTAAGGGTGGGCCTCTCTTCTCAGAAATTTTGAAGAATTGGAAAGATGAA      180
                 ************************************************************

NM_174086.1      AGTGACAAAAAAATTATTCAGAGCCAAATTGTCTCCTTCTACTTCAAACTCTTTGAAAAC      240
H                AGTGACAAAAAAATTATTCAGAGCCAAATTGTCTCCTTCTACTTCAAACTCTTTGAAAAC      240
P                AGTGACAAAAAAATTATTCAGAGCCAAATTGTCTCCTTCTACTTCAAACTCTTTGAAAAC      240
                 ************************************************************

NM_174086.1      CTCAAAGATAACCAGGTCATTCAAAGGAGCATGGATATCATCAAGCAAGACATGTTTCAG      300
H                CTCAAAGATAACCAGGTCATTCAAAGGAGCATGGATATCATCAAGCAAGACATGTTTCAG      300
P                CTCAAAGATAACCAGGTCATTCAAAGGAGCATGGATATCATCAAGCAAGACATGTTTCAG      300
                 ************************************************************

NM_174086.1      AAGTTCTTGAATGGCAGCTCT        321
H                AAGTTCTTGAATGGCAGCTCT        321
P                AAGTTCTTGAATGGCAGCTCT        321
                 *********************
```

Figure S6: Representative Sequence alignment of *IFN-γ* gene (321-bp) between healthy (H), and pneumonic (P) calves.

```
NM_174643.1     ACGGAGCTCGGCAGAGCAGCAAAGCTGGCACCAGAATTTGCCAAGAGAAATGTCAAGATG     60
H               ACGGAGCTCGGCAGAGCAGCAAAGCTGGCACCAGAATTTGCCAAGAGAAATGTCAAGATG     60
P               ACGGAGCTCGGCAGAGCAGCAAAGCTGGCACCAGAATTTGCCAAGAGAAATGTCAAGATG     60
                ************************************************************

NM_174643.1     ATTGCTCTTTCCATAGACAGTGTGGAAGACCATCTTGCATGGAGCAAGGATATCAATGCT     120
H               ATTGCTCTTTCCATAGACAGTGTGGAAGACCATCTTGCATGGAGCAAGGATATCAATGCT     120
P               ATTGCTCTTTCCATAGACAGTGTGGAAGACCATCTTGCATGGAGCAAGGATATCAACGCT     120
                ******************************************************** ***

NM_174643.1     TACAATGGTGAAGAGCCCACAGAAAAGTTACCTTTTCCCATCATTGATGATAAGAATCGG     180
H               TACAATGGTGAAGAGCCCACAGAAAAGTTACCTTTTCCCATCATTGATGATAAGAATCGG     180
P               TACAATGGTGAAGAGCCCACAGAAAAGTTACCTTTTCCCATCATTGATGATAAGAATCGG     180
                ************************************************************

NM_174643.1     GACCTTGCCATCCAGTTGGGCATGCTGGACCCAGCAGAGAAAGATGAAAAGGGCATGCCT     240
H               GACCTTGCCATCCAGTTGGGCATGCTGGACCCAGCAGAGAAAGATGAAAAGGGCATGCCT     240
P               GACCTTGCCATCCAGTTGGGCATGCTGGACCCAGCAGAGAAAGACGAAAAGGGCATGCCT     240
                ******************************************** ***************

NM_174643.1     GTGACTGCTCGTGTGGTGTTTATTTTTGGTCCTGATAAGAAACTGAAACTGTCCATCCTC     300
H               GTGACTGCTCGTGTGGTGTTTATTTTTGGTCCTGATAAGAAACTGAAACTGTCCATCCTC     300
P               GTGACTGCTCGTGTGGTGTTTATTTTTGGTCCTGATAAGAAACTGAAACTGTCCATCCTC     300
                ************************************************************

NM_174643.1     TACCCAGCTACCACTGGCAGGAACTTTGATGAGATTCTCAGAGTAATTATCTCTCTCCAG     360
H               TACCCAGCTACCACTGGCAGGAACTTTGATGAGATTCTCAGAGTAATTATCTCTCTCCAG     360
P               TACCCAGCTACCACTGGCAGGAACTTTGATGAGATTCTCAGAGTAATTATCTCTCTCCAG     360
                ************************************************************

NM_174643.1     CTGACGGCAGAAAAGAGGGTGGCCACCCCGGTTGACTGGAAGAATGGGGACAGCGTGATG     420
H               CTGACGGCAGAAAAGAGGGTGGCCACCCCGGTTGACTGGAAGAATGGGGACAGCGTGATG     420
P               CTGACGGCAGAAAAGAGGGTGGCCACCCCGGTTGACTGGAAGAATGGGGACAGCGTGATG     420
                ************************************************************

NM_174643.1     GTCCTTCCAACCAT     434
H               GTCCTTCCAACCAT     434
P               GTCCTTCCAACCAT     434
                **************
```

Figure S7: An example of the alignment of the *PRDX6* gene (434-bp) between calves with pneumonia (P) and healthy (H).

```
NM_001142967.1      GGTGTCAGACACATCACGTTCGTGGACAATGCCAAGATCTCCTACTCTAACCCTGTGAGG    60
P                   GGTGTCAGACACATCACGTTCGTGGACAATGCCAAGATCTCCTACTCCAACCCTGTGAGG    60
H                   GGTGTCAGACACATCACGTTCGTGGACAATGCCAAGATCTCCTACTCTAACCCTGTGAGG    60
                    *********************************************** ************

NM_001142967.1      CAGCCTCTGTATGAATTTGAAGATTGCCTGGCGGGTGGGAAGCCCAAGGCCCTGGCAGCC    120
P                   CAGCCTCTGTATGAATTTGAAGATTGCCTGGCGGGTGGGAAGCCCAAGGCCCTGGCAGCC    120
H                   CAGCCTCTGTATGAATTTGAAGATTGCCTGGCGGGTGGGAAGCCCAAGGCCCTGGCAGCC    120
                    ************************************************************

NM_001142967.1      GCAGACCGGCTGCAGAAGATATTCCCCGGAGTGAACGCCAGAGGGTTCAACATGAGCATC    180
P                   GCAGACCGGCTGCAGAAGATATTCCCCGGAGTGAACGCCAGAGGGTTCAACATGAGCATC    180
H                   GCAGACCGGCTGCAGAAGATATTCCCCGGAGTGAACGCCAGAGGGTTCAACATGAGCATC    180
                    ************************************************************

NM_001142967.1      CCCATGCCGGGGCACCCCGTGAACTTCTCCAGCGTCACCCTGGAGCAGGCCCGCAGGGAC    240
P                   CCCATGCCGGGGCACCCCGTGAACTTCTCCAGCATCACCCTGGAGCAGGCCCGCAGGGAC    240
H                   CCCATGCCGGGGCACCCCGTGAACTTCTCCAGCGTCACCCTGGAGCAGGCCCGCAGGGAC    240
                    ********************************* **************************

NM_001142967.1      GTGGAACAGCTGGAGCAGCTCATTGACAGCCACGACGTCGTCTTCCTGTTGATGGACACC    300
P                   GTGGAACAGCTGGAGCAGCTCATTGACAGCCACGACGTCGTCTTCCTGTTGATGGACACC    300
H                   GTGGAACAGCTGGAGCAGCTCATTGACAGCCACGACGTCGTCTTCCTGTTGATGGACACC    300
                    ************************************************************

NM_001142967.1      CGGGAGAGCCGGTGGCTGCCTGCCGTCATCGCCGCGAGCAAGAGAAAGCTGGTCATCAAT    360
P                   CGGGAGAGCCGGTGGCTGCCTGCCGTCATCGCTGCGAGCAAGAGAAAGCTGGTCATCAAT    360
H                   CGGGAGAGCCGGTGGCTGCCTGCCGTCATCGCCGCGAGCAAGAGAAAGCTGGTCATCAAT    360
                    ******************************** ***************************

NM_001142967.1      GCTGCCTTGGGATTTGACACCTTCGTTGTCATGAGACACGGCCTGAAGAAGCCCAG        416
P                   GCTGCCTTGGGATTTGACACCTTCGTTGTCATGAGACACGGCCTGAAGAAGCCCAG        416
H                   GCTGCCTTGGGATTTGACACCTTCGTTGTCATGAGACACGGCCTGAAGAAGCCCAG        416
                    ********************************************************
```

Figure S8: An example of the alignment of the *ATG7* gene (416-bp) between calves with pneumonia (P) and healthy (H).

```
XM_005221658.4      ACGCGCGGCCTGCCCGGGGGCGCCAGGTGCTTTGGGGTGCGGACCTCGCCGACCGGGGAG    60
H                   ACGCGCGGCCTGCCCGGGGGCGCCAGGTGCTTTGGGGTGCGGACCTCGCCGACCGGGGAG    60
P                   ACGCGCGGCCTGCCCGGGGGCGCCAGGTGCTTTGGGGTGCGGACCTCGCCGACCGGGGAG    60
                    ************************************************************

XM_005221658.4      AAGGTCACGCACACCGGCCAGGTTTATGACGATGGAGACTACAGGAAAGTTCGATTTGTA    120
H                   AAGGTCACGCACACCGGCCAGGTTTATGACGATGGAGACTACAGGAAAGTTCGATTTGTA    120
P                   AAGGTCACGCACACCGGCCAGGTTTATGACGATGGAGACTACAGGAAAGTTCGATTTGTA    120
                    ************************************************************

XM_005221658.4      GGTCGTCAGAAAGAGGTGAACGAGAACTTTGCCATCGATCTGATAGCGGAGCAGCCCGTG    180
H                   GGTCGTCAGAAAGAGGTGAACGAGAACTTTGCCATCGATCTGATAGCGGAGCAGCCCGTG    180
P                   GGTCGTCAGAAAGAGGTGAACGAGAACTTTGCCATCGATCTGATAGCGGAGCAGCCCGTG    180
                    ************************************************************

XM_005221658.4      AGCCAAGTTGGGAGTCGGGTGATATCGTGCGACGGCGGCGGGGGGGCCCTGGGCCACCCC    240
H                   AGCCAAGTTGGGAGTCGGGTGATATCGTGCGACGGCGGCGGGGGGGCCCTGGGCCACCCC    240
P                   AGCCAAGTTGGGAGTCGGGTGATATCGTGCGACGGCGGCGGGGGGGCCCTGGGCCACCCC    240
                    ************************************************************

XM_005221658.4      CGAGTGTACATAAACCTGGTAATGTGCTGTCCAGCCCCCAGCCCCAGCCCCGGTTCCTGT    300
H                   CGAGTGTACATAAACCTGGTAATGTGCTGTCCAGCCCCCAGCCCCAGCCCCGGTTCCTGT    300
P                   CGAGTGTACATAAACCTGGTAATGTGCTGTCCAGCCCCCAGCCCCAGCCCCGGTTCCTGT    300
                    ************************************************************

XM_005221658.4      CCTGTTCTGTCCCCGCTGGCCGCACACGCTCGGCTCGGCCCTGCACACGAGTCGTCCGCC    360
H                   CCTGTTCTGTCCCCGCTGGCCGCACACGCTCGGCTCGGCCCTGCACACGAGTCGTCCGCC    360
P                   CCTGTTCTGTCCCCGCTGGCCGCACACGCTCGGCTCGGCCCTGCACACGAGTCATCCGCC    360
                    ***************************************************** ******

XM_005221658.4      AGGGCTGAGCAGAGGGCTGGCACGCACAGAGCTTCCAGGACAGGA         405
H                   AGGGCTGAGCAGAGGGCTGGCACGCACAGAGCTTCCAGGACAGGA         405
P                   AGGGCTGAGCAGAGGGCTGGCACGCACAGAGCTTCCAGGACAGGA         405
                    *********************************************
```

Figure S9: Representative Sequence alignment of *NDUFS6* gene (405-bp) between healthy (H), and pneumonic (P) calves.

```
NM_001304775.1      CACCTCTGCCTGCTTATCTGGCTCTCCCTGAATGTCCTGCTTTTCTGGAAAACCTTTCTG   60
H                   CACCTCTGCCTGCTTATCTGGCTCTCCCTGAATGTCCTGCTTTTCTGGAAAACCTTTCTG   60
P                   CACCTCTGCCTGCTTATCTGGCTCTCCCTGAATGTCCTGCTTTTCTGGAAAACCTTTCTG   60
                    ************************************************************

NM_001304775.1      CTGTATAACCAAGGGCCAGAGTATCACTACCTCCACGAGATGCTGGGGCTAGGATTGTGT   120
H                   CTGTATAACCAAGGGCCAGAGTATCACTACCTCCACGAGATGCTGGGGCTAGGATTGTGT   120
P                   CTGTATAACCAAGGGCCAGAGTATCACTACCTCCACGAGATGCTGGGGCTAGGATTGTGT   120
                    ************************************************************

NM_001304775.1      CTAAGCAGAGCCTCTGCATCTGTTCTTAACCTCAACTGCAGCCTTATCCTCTTACCCATG   180
H                   CTAAGCAGAGCCTCTGCATCTGTTCTTAACCTCAACTGCAGCCTTATCCTCTTACCCATG   180
P                   CTAAGCAGAGCCTCTGCATCTGTTCTTAACCTCAACTGCAGCCTTATCCTCTTACCCATG   180
                    ************************************************************

NM_001304775.1      TGCCGCACCCTCCTGGCTTTCCTTCGAGGATCGCAGAAGGTTCCAAGCCGGAGAACCAGA   240
H                   TGCCGCACCCTCCTGGCTTACCTTCGAGGATCACAGAAGGTTCCAAGCCGGAGAACCAGA   240
P                   TGCCGCACCCTCCTGGCTTTCCTTCGAGGATCGCAGAAGGTTCCAAGCCGGAGAACCAGA   240
                    ******************* ************ ***************************

NM_001304775.1      AGATTGTTGGATAAAAGCAGAACATTCCACATAACCTGTGGTGTGACTATCTGTATTTTC   300
H                   AGATTGTTGGATAAAAGCAGAACATTCCACATAACCTGTGGTGTGACTATCTGTATTTTC   300
P                   AGATTGTTGGATAAAAGCAGAACATTCCACATAACCTGTGGTGTGACTATCTGTATTTTC   300
                    ************************************************************

NM_001304775.1      TCAGGTGTGCACGTGGCCGCCCACCTGGTTAATGCCCTCAACTTCTCAGTGAACTACCGC   360
H                   TCAGGTGTGCACGTGGCCGCCCACCTGGTTAATGCCCTCAACTTCTCAGTGAACTACCGC   360
P                   TCAGGTGTGCACGTGGCCGCCCACCTGGTTAATGCCCTCAACTTCTCAGTGAACTACCGC   360
                    ************************************************************

NM_001304775.1      GAGGATTTTGTCGAACTGAATGCAGCAAGATACCGA                 396
H                   GAGGATTTTGTCGAACTGAATGCAGCAAGATACCGA                 396
P                   GAGGATTTTGTCGAACTGAATGCAGCAAGATACCGA                 396
                    ************************************
```

Figure S10: An example of the alignment of the *NOX4* gene (396-bp) between calves with pneumonia (P) and healthy (H).
